# Influence of Induced Blood Pressure Variability on the Assessment of Cerebral Autoregulation in Patients after Cardiac Arrest

**DOI:** 10.1155/2018/8153241

**Published:** 2018-06-06

**Authors:** J. M. D. van den Brule, C. R. van Kaam, J. G. van der Hoeven, J. A. H. R. Claassen, C. W. E. Hoedemaekers

**Affiliations:** ^1^Department of Intensive Care, Radboud University Nijmegen Medical Centre, Nijmegen, Netherlands; ^2^Department of Geriatrics, Radboud University Nijmegen Medical Centre, Nijmegen, Netherlands

## Abstract

**Objective:**

To determine if increasing variability of blood pressure influences determination of cerebral autoregulation.

**Methods:**

A prospective observational study was performed at the ICU of a university hospital in the Netherlands. 13 comatose patients after cardiac arrest underwent baseline and intervention (tilting of bed) measurements. Mean flow velocity (MFV) in the middle cerebral artery and mean arterial pressure (MAP) were measured. Coefficient of variation (CV) was used as a standardized measure of dispersion in the time domain. In the frequency domain, coherence, gain, and phase were calculated in the very low and low frequency bands.

**Results:**

The CV of MAP was significantly higher during intervention compared to baseline. On individual level, coherence in the VLF band changed in 5 of 21 measurements from unreliable to reliable and in 6 of 21 measurements from reliable to unreliable. In the LF band 1 of 21 measurements changed from unreliable to reliable and 3 of 21 measurements from reliable to unreliable. Gain in the VLF and LF band was lower during intervention compared to baseline.

**Conclusions:**

For the ICU setting, more attention should be paid to the exact experimental protocol, since changes in experimental settings strongly influence results of estimation of cerebral autoregulation.

## 1. Introduction

Postanoxic encephalopathy is a common phenomenon after cardiac arrest and causes high mortality and morbidity [1]. Adapting the cerebral blood flow (CBF) to the cerebral metabolic demand improves cerebral recovery after cardiac arrest.

Cerebral autoregulation (CA) describes the process of cerebral vasodilation and vasoconstriction to maintain a stable CBF over a wide range of perfusion pressures. This adjustment of CBF to changes in cerebral perfusion pressure is directed by central regulation-mechanisms [2, 3] and by a local adaptation using myogenic vasoconstriction [4, 5]. In the low frequency band (LF, 0.07–0.2 Hz) blood pressure variations are provoked by sympathetic modulation of vascular tone [6]. Local vascular myogenic changes in blood pressure occur in the LF and very low frequency bands (VLF, 0.02–0.07 Hz) [7]. In the high frequency band (HF, 0.2–0.5 Hz) nitric oxide (NO) affects cardiovascular variability in animals [8]. Cerebral autoregulation is disturbed in a large proportion of patients after cardiac arrest [9, 10]. Disturbed autoregulation in the early phase after cardiac arrest is strongly associated with unfavourable outcome [9].

After cardiac arrest, the spontaneous variability of arterial pressure is significantly lower compared to age- and sex-matched control patients [11]. In addition, spontaneous variability in the mean flow velocity (MFV) in the middle cerebral artery as measured by transcranial Doppler (TCD) is also reduced. MFV variability restores towards normal values in survivors after cardiac arrest, while variability continues to decline in patients who do not survive [11].

For the estimation of dynamic CA, transfer function analysis (TFA) is considered the gold standard. TFA describes the dynamic relationship between blood pressure (input signal) and CBF (output signal). This technique relies on spontaneous or induced fluctuations in MFV and mean arterial pressure (MAP). Reduced variability in the input signal will strongly reduce the reliability of the resulting output signal [12]. Recently, a white paper was published by the Cerebral Autoregulation Research Network (CARNet, http://www.car-net.org) with recommendations to improve the standardization and settings for TFA applications in studies of dynamic autoregulation [12]. Because of a lack of evidence in this matter, this paper could not formulate a recommendation regarding the minimum variability of the input signal that is required for reliable estimation of dynamic autoregulation. As a result, low variability may not be regarded as a criterion to reject or at least critically appraise CA data.

The main objective of our study was to determine if an increase in variability of the blood pressure signal influences determination of the state of autoregulation both in the time and in the frequency domain.

## 2. Materials and Methods

### 2.1. Study

A prospective observational study was performed at the intensive care unit (ICU) of a tertiary care university hospital in Nijmegen, the Netherlands.

### 2.2. Population

We studied 13 comatose patients admitted to the ICU after an out-of-hospital cardiac arrest. Inclusion criteria were age ≥ 18 years and a Glasgow Coma Score ≤ 8 after return of spontaneous circulation. Patients were included after written informed consent and approval of the protocol by the local Institutional Review Board (document number 2015-1567, 52259.091.15).

Exclusion criteria were an irregular heart rhythm, no transtemporal bone window, pregnancy, thrombolytic therapy, refractory cardiogenic shock, intra-aortic balloon pump, a life expectancy ≤ 24 hours and known carotid artery stenosis, or signs of carotid artery stenosis on physical examination or ultrasound.

### 2.3. Patient Management

The patients were treated according to the local protocol as described in a previous manuscript [11]. In short, this included mild therapeutic hypothermia 32–34°C for 24 hours, followed by passive rewarming to 37°C. All patients were sedated and sedation was stopped as soon as the temperature reached 36°C [11]. All patients were intubated and mechanically ventilated to obtain a PaO_2_ > 75 mmHg and a PaCO_2_ 34–41 mmHg [11]. An arterial catheter was used for monitoring of arterial blood pressure (ABP) and sampling of blood. According to this local protocol, MAP was maintained 80–100 mmHg [11].

### 2.4. Data Collection

Demographic, prehospital, and clinical data were collected. MFV in the middle cerebral artery was measured by TCD through the temporal window with a 2-Mhz probe (Multi-Dop T Digital, Compumedics DWL, Singen, Germany) as described in a previous manuscript [11]. All measurements were performed by two investigators (J.B. and C.H.). A 30-minute window of cerebral blood flow velocity (CBFV) and ABP was simultaneously recorded on a laptop computer and stored on a hard disk with a sample rate of 200 Hz by an A/D converter (NI USB-6211, National Instrument, Austin, TX, USA).

### 2.5. Intervention

Baseline recordings of continuous ABP and CBFV were performed with the patient at rest, with the head of bed elevated at 30 degrees. The intervention consisted of repeated changes in the position of the bed from horizontal to maximum 30 degrees Trendelenburg and 30 degrees anti-Trendelenburg (period 6 minutes), thereby inducing low frequency blood pressure fluctuations. The blood pressure transducer was attached to the bed at right atrium level and its position changed simultaneously with the position of the patient. The patient was moved for 15 seconds from Trendelenburg to non-Trendelenburg and remained in this position for 45 seconds. A total of 3 Trendelenburg and 3 non-Trendelenburg positions were measured during the 6 minutes of testing. Changes were timed and recorded. During the measurements, PaO_2_ and PaCO_2_ were within normal ranges and stable.

Measurements were performed on admission to the ICU and at 24, 48, and 72 hours.

### 2.6. Data Analysis

CBFV and ABP data were analyzed using custom-written MATLAB scripts (Matlab R2014b, The MathWorks Inc., Massachusetts, USA), as described in a previous manuscript [11]. From these ABP and CBFV signals, 5-minute segments of baseline and intervention data were selected based on the least amount of artefacts [11]. MAP and MFV were acquired by filtering ABP and CBFV with a third-order zero phase-lag Butterworth filter with a cut-off frequency of 0.5 Hz [11]. By averaging these 5-minute windows of the MAP and MFV signals, mean values of MAP and MFV were acquired [11]. For transfer function analysis, the CARNet TFA MATLAB script was used (available on http://www.car-net.org).

To validate our analysis, an external expert (J.C.) went through the analysis step by step to check whether this analysis had been performed in agreement with the recommendations in the white paper on transfer function analysis of the International Cerebral Autoregulation Research Network [12].

### 2.7. Blood Pressure Variation

Coefficient of variation (CV) was used as a standardized measure of dispersion for both MAP and MFV in the time domain. CV was defined as the standard deviation of the signal divided by the mean of the signal and was calculated from all filtered signals. This way, the variation is expressed in percentage of the mean.

In the frequency domain, the average spectral power of MAP and MFV was calculated as a measure of variation in the very low (VLF, 0.02–0.07 Hz) and low (LF, 0.07–0.2 Hz) frequency bands.

### 2.8. Cerebral Autoregulation

CA was calculated in the time domain and frequency domain. In the time domain, CA was calculated by the mean flow velocity index (Mx) as a Pearson's correlation coefficient between 10-second averages of ABP and CBFV over the 5-minute time window [13]. A cut-off value of 0.3 was chosen to indicate absence or presence of autoregulation [14].

In the frequency domain, TFA was performed according to the recommendations by the international Cerebral Autoregulation Research Network (CARNet) [12]. In short, the 5-minute segments of MFV and MAP signals were resampled to 10 Hz. Both auto- and cross-spectra were estimated based on Welch's method using a sliding Hanning window of 100 seconds' window length with 50% overlap. Spectral smoothing was applied by using a triangular moving average window. Gain and phase were only calculated when coherence was above the critical coherence threshold based on the 95% confidence interval. The cut-off value was 0.34. The TFA coherence, gain, and phase were calculated for the very low (VLF, 0.02–0.07 Hz) and low (LF, 0.07–0.2 Hz) frequency bands.

### 2.9. Statistical Analysis

Statistical analysis was performed using GraphPad Prism version 5.0 (GraphPad Software, La Jolla, CA). Data were checked for Gaussian distribution by a Kolmogorov-Smirnov test. Paired, normal distributed data were analyzed with a paired* t*-test and presented as mean with standard deviation. Paired non-Gaussian distributed data were analyzed with a Wilcoxon matched-pairs signed rank test and presented as median with 25th and 75th percentile.

A *p* value of <0.05 was considered to indicate significance.

## 3. Results

### 3.1. Demographic and Clinical Data

We included 13 comatose patients successfully resuscitated after cardiac arrest and treated with mild therapeutic hypothermia. The median age was 61 [50–65] years. Twelve patients had ventricular fibrillation (VF) or ventricular tachycardia (VT) as initial rhythm, and 1 patient initially had a pulseless electrical activity (PEA). Four patients died in the ICU, all because of severe postanoxic brain damage. The clinical and laboratory data on admission are summarized in Table 1.

### 3.2. Mean Arterial Blood Pressure Variation and Mean Flow Velocity Variation in the Time Domain

**Figure 1:**
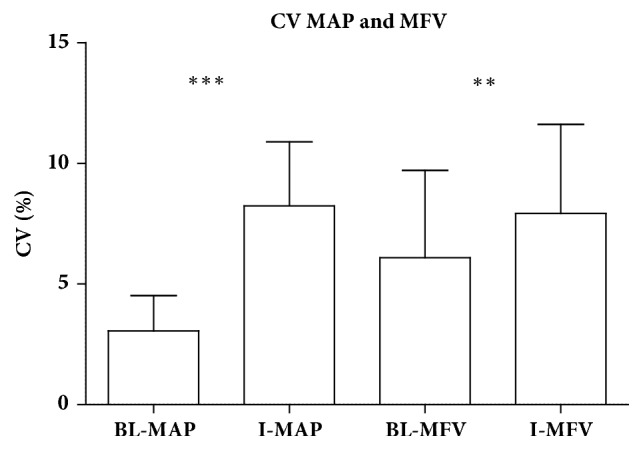
CV of MAP and MVF after cardiac arrest in baseline position and during intervention. BL: baseline; CV: coefficient of variation; I: intervention; MAP: mean arterial pressure; MVF: mean flow velocity.

Tilting of the bed induced a significant increase in CV of MAP from 3.056 ± 1.464 at baseline to 8.238 ± 2.646 during intervention (*p* < 0.0001) (Figure 1). The CV of MFV after cardiac arrest was also significantly higher during the intervention (7.571 [6.124–9.176]) compared to baseline (6.090 ± 3.613) (*p* = 0.0028) (Figure 1).

### 3.3. Cerebral Autoregulation

#### 3.3.1. Time Domain

**Figure 2:**
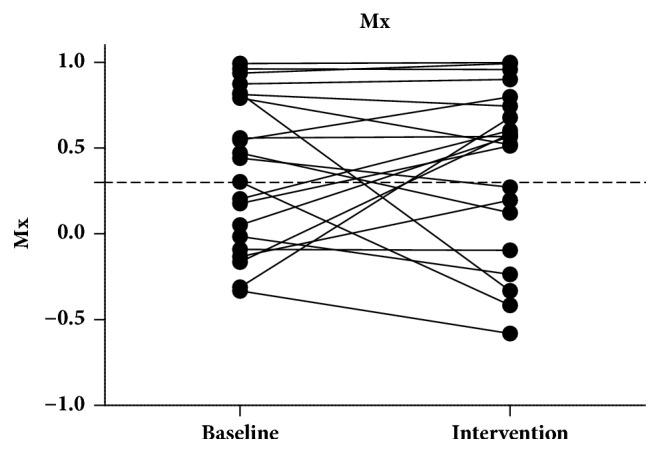
Mx after cardiac arrest in baseline position and during intervention. Five of the 21 measurements changed from intact to affected CA and 4 of the 21 measurements changed from affected to intact CA. Mx: mean flow velocity index.

On group level, Mx did not change during intervention (0.5669 [0.0132–0.7712]) compared to baseline (0.3757 ± 0.4473) (*p* = 0.6776) (Figure 2). On individual level, in 9 of the 21 measurements (43%) interpretation of the CA changed during intervention. Five measurements changed from intact to impaired CA and 4 measurements changed from impaired to intact CA.

#### 3.3.2. Frequency Domain

**Figure 3:**
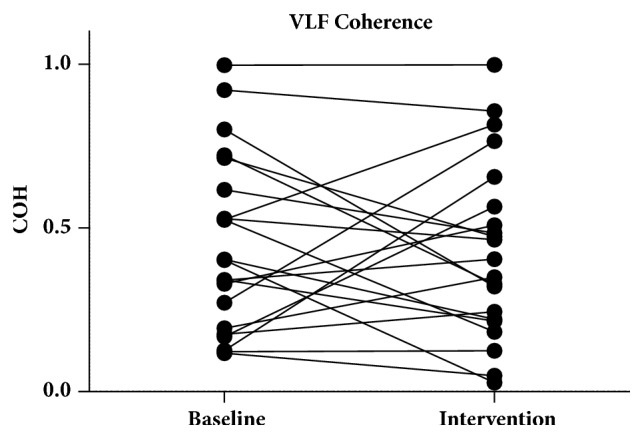
Coherence in the VLF band. Five of the 21 measurements changed from unreliable to reliable and 6 of the 21 measurements changed from reliable to unreliable. VLF: very low frequency.

**Figure 4:**
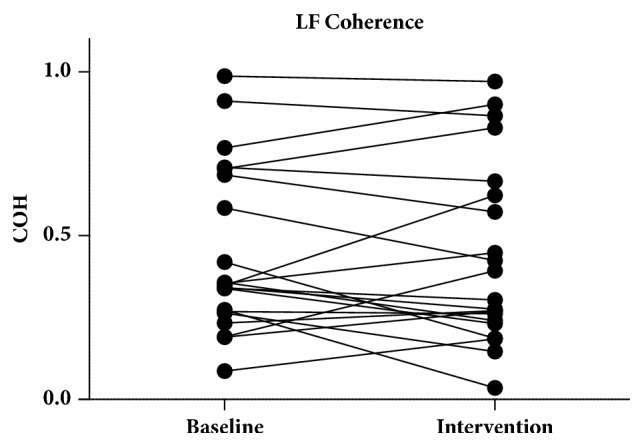
Coherence in the LF band. One of the 21 measurements changed from unreliable to reliable and 3 of the 21 measurements changed from reliable to unreliable. LF: low frequency.

There were no significant changes on a group level in coherence between baseline (0.4449 ± 0.2692) and intervention (0.4313 ± 0.2702) in the VLF band (*p* = 0.8281) (Figure 3). There were also no changes in coherence in the LF band between baseline (0.3476 [0.2655–0.6949]) and intervention (0.3043 [0.2361–0.6444]) (*p* = 0.3265) (Figure 4). On an individual level, in the VLF band, 5 of the 21 measurements changed from unreliable to reliable and 6 of the 21 measurements changed from reliable to unreliable. In the LF band 1 of the 21 measurements changed from unreliable to reliable and 3 of the 21 measurements changed from reliable to unreliable.

**Figure 5:**
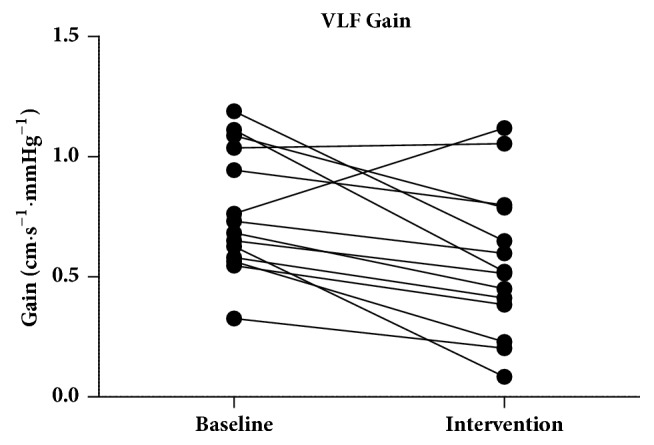
The gain in the VLF band during intervention was lower compared to the gain in the VLF baseline (*p* = 0.0058). VLF: very low frequency.

**Figure 6:**
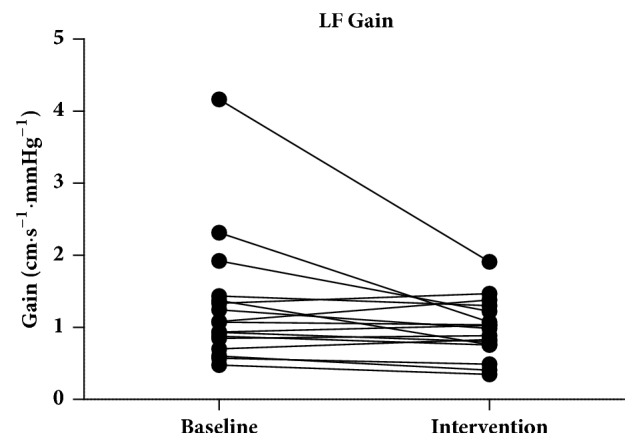
The gain in the LF band during intervention was lower compared to the gain in the LF band baseline (*p* = 0.0523). LF: low frequency.

The gain in the VLF band during intervention (0.5578 ± 0.3046) was lower compared to the gain in the VLF baseline (0.7746 ± 0.2579) (*p* = 0.0058) (Figure 5). The gain in the LF band during intervention (0.9817 ± 0.4007) was lower compared to the gain in the LF band baseline (1.075 [0.7757–1.405]) (*p* = 0.0523) (Figure 6).

**Figure 7:**
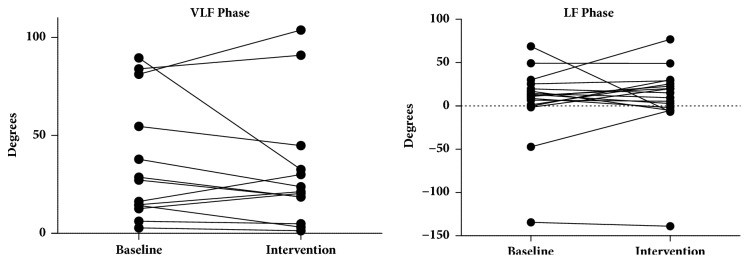
There were no changes in phase in the VLF and LF band during intervention compared to the phase in baseline position (*p* = 0.4548 and 0.570, respectively). LF: low frequency; VLF: very low frequency.

There were no changes in phase in the VLF and LF band during intervention (21.29 [11.73–38.64] and 14.96 [−3.546–27.16], respectively) compared to the phase in baseline position (36.12 ± 31.01 and 12.00 [0.1032–22.62], respectively) (*p* = 0.4548 and 0.570, respectively) (Figure 7).

## 4. Discussion

Amplification of blood pressure variability changed the interpretation of CA as measured by Mx and TFA. This difference in test results is not related to physiological changes in CA, as both baseline and intervention were performed at the same time-point after cardiac arrest. This difference in test results is most likely related to bias induced by very low variability in the blood pressure and MFV signal at baseline, which renders estimation of CA with these methods unreliable.

For the estimation of dynamic CA, TFA is considered the gold standard. The white paper on TFA methodology provides recommendations on the optimal cut-off values of coherence and parameter settings [12]. No recommendations are available for the optimal experimental or clinical protocol. High quality of both the ABP and MFV signal are essential for reliable estimation of CA [15]. Poor quality of the temporal bone insonation conditions or poor quality of the arterial or MFV signal can result in considerable bias of the TFA results. Quality of both signals was adequate in our study and did not explain the intervention induced changes in TFA and Mx in our population.

The optimal recording time of signals for autoregulation to stabilize is largely unknown. Recording time for the autoregulatory index Mx significantly influences validity of the measurements. Mx calculated on intervals shorter than 6 minutes is at risk for changes due to insufficient stabilization of the signal [16]. The Mx data were determined from a 5-minute time window, possibly influencing the validity of the Mx.

The white paper recommends a minimum window length for TFA analysis of at least 100 seconds [12]. This is based on the fact that window lengths shorter than 75 seconds resulted in increased bias [12]. Recording time of the TFA signals at baseline and during intervention in our study was sufficiently long to avoid bias related to time restriction.

The extent to which TFA outcomes are influenced by quality of the signals differs between different parameters. In our study, increased variability of signals resulted in decrease in gain in both the LF and VLF bands, whereas phase remained stable. This is most likely related to the fact that gain reflects the strength of the autoregulation, which may be changed by increased amplitude of the signal. In contrast, phase is related to velocity of dynamic changes in autoregulation. Since augmentation of the variability is unlikely to change the timing of the arterial and cerebral pulses, phase may remain unchanged during the intervention.

The use of the coherence function is recommended to identify conditions where estimates of gain and phase may not be reliable. The intervention changed the level of coherence from unreliable to reliable in a substantial portion of patients, but the reverse (change from reliable to unreliable) occurred in a similar proportion of patients. Since the variability was extremely low, the input signal may have been too low for adequate determination of TFA coherence at baseline.

This was supported by visual inspection of the raw data and linking this with individual outcomes of TFA. Visual inspection of the raw signals can easily identify whether oscillations in MAP and CBFV are present and whether these oscillations are temporally related. In the few patients who showed variability in both MAP and CBFV, the results of TFA appeared reliable, with higher phase values in VLF than in LF and lower gain values in VLF than in LF. In contrast, when TFA was performed in patients with low MAP variability, these normal patterns were not observed, even if coherence was above the threshold.

In an earlier study we proved that the spontaneous variability of the MAP remained low during the entire study period after cardiac arrest [11]. In the current study we demonstrated a significant increase in CV of MAP during tilting of the bed compared to the resting position. Previous literature showed an improvement of reproducibility of the autoregulation coefficients by inducing blood pressure variability due to a sit-to-stand manoeuvre [17, 18]. However, those manoeuvres lead to consistent oscillatory changes that enhance reliability, in contrast with bed tilting.

This study has a number of limitations. We performed an observational study in a relatively small population. Tilting of the bed may result in changes in quality of the recorded signal, such as loss of signal, motion artefacts, and baseline drifts. Reanalysis of the raw signals however was carefully performed by an independent expert (J.C.), using only good quality signals and verifying correct TFA settings, but this did not alter the outcome. Changes in the bed position may influence the pressure in the veins draining the cerebral venous blood. These changes in venous outflow pressure may influence cerebral perfusion and thus the validity of the autoregulation measurements.

## 5. Conclusions

The white paper on TFA methodology currently provides no recommendations about minimal necessary blood pressure variation to perform a reliable TFA. Given the results of this study, addition of minimal necessary variation of input signals to the white paper on TFA methodology may improve the validity of TFA measurements. For the ICU setting, more attention should be paid to the exact experimental protocol, since changes in experimental settings strongly influence results of estimation of CA. The optimal level of variation of the signals will be difficult to establish because of the lack of golden standards of autoregulation for comparison of the results. Computer modelling may be helpful to establish the effects of changes in different parameters on the measurements of autoregulation.

## Figures and Tables

**Table 1:** Clinical and laboratory data of cardiac arrest patients on admission. PEA: pulseless electrical activity; VF: ventricular fibrillation; VT: ventricular tachycardia.

Characteristics	
Age (years)	61 [50–65]
Male (*n*, %)	12/13 (92%)
Initial rhythm VT/VF (*n*, %)	12/13 (92%)
Initial rhythm PEA (*n*, %)	1/13 (8%)
SAPS 2	58 [42–77]
APACHE 2	27 [18–30]
pH	7.01 [6.94–7.27]
Lactate (mmol/L)	8.5 [7.0–13.3]
PaCO2 (mmHg)	7.0 [6.2–9.5]

## Abbreviations

ABP: Arterial blood pressure
CA: Cerebral autoregulation
CBF: Cerebral blood flow
CBFV: Cerebral flow velocity
CV: Coefficient of variation
ICU: Intensive care unit
MAP: Mean arterial pressure
Mx: Mean flow velocity index
NO: Nitric oxide
PEA: Pulseless electrical activity
TCD: Transcranial Doppler
TFA: Transfer function analysis
VF: Ventricular fibrillation
VT: Ventricular tachycardia.
